# A Novel Matrix Protein Hic31 from the Prismatic Layer of *Hyriopsis Cumingii* Displays a Collagen-Like Structure

**DOI:** 10.1371/journal.pone.0135123

**Published:** 2015-08-11

**Authors:** Xiaojun Liu, Shimei Zeng, Shaojian Dong, Can Jin, Jiale Li

**Affiliations:** 1 Key Laboratory of Freshwater Aquatic Genetic Resources, Shanghai Ocean University, Ministry of Agriculture, Shanghai, China; 2 Shanghai Engineering Research Center of Aquaculture (ZF1206), Shanghai Ocean University, Shanghai, China; 3 Shanghai University Knowledge Service Platform, Shanghai Ocean University Aquatic Animal Breeding Center (ZF1206), Shanghai Ocean University, Shanghai, China; 4 E-Institute of Shanghai Universities, Shanghai Ocean University, Shanghai, China; Temasek Life Sciences Laboratory, SINGAPORE

## Abstract

In this study, we clone and characterize a novel matrix protein, hic31, from the mantle of *Hyriopsis cumingii*. The amino acid composition of hic31 consists of a high proportion of Glycine residues (26.67%). Tissue expression detection by RT-PCR indicates that hic31 is expressed specifically at the mantle edge. *In situ* hybridization results reveals strong signals from the dorsal epithelial cells of the outer fold at the mantle edge, and weak signals from inner epithelial cells of the same fold, indicating that hic31 is a prismatic-layer matrix protein. Although BLASTP results identify no shared homology with other shell-matrix proteins or any other known proteins, the hic31 tertiary structure is similar to that of collagen I, alpha 1 and alpha 2. It has been well proved that collagen forms the basic organic frameworks in way of collagen fibrils and minerals present within or outside of these fibrils. Therefore, hic31 might be a framework-matrix protein involved in the prismatic-layer biomineralization. Besides, the gene expression of hic31 increase in the early stages of pearl sac development, indicating that hic31 may play important roles in biomineralization of the pearl prismatic layer.

## Introduction

Many living organisms are capable of converting inorganic ions into solid minerals through a dynamic physiological process called biomineralization [1, 2]. This process allows the formation of many external and internal hard tissues (e.g. shells, pearls, and bones) that display a wide range of functions [3]. Among biomineralization products, the mollusk shell and pearl (especially the nacre of shells or pearls as a non-human organic-mineral biomaterial) becomes the focus of biomaterial and aquatic research due to their highly-ordered microstructure and superior mechanical properties [2, 4]. The nacre is usually comprised of 95% calcium carbonate and accounts for only 0.1%-5% of the organic matrix, of which the organic matrix are densely packed with proteins, polysaccharides, and lipids [5]. These macro-molecules are secreted by the polarized mantle characterized by three folds among bivalves. The outer epithelial cells in the outer folds of different regions are responsible for nacre deposition and secretion of prism precursors. In general, the outer epithelium of the edge in the outer folds is always related to the formation of prismatic layer, while the dorsal region is always involved in nacreous layer formation. Until now, researchers has revealed that various phases, including nucleation, crystallization, crystal orientation, and crystal morphology, can be influenced by proteins extracted from shell through interactions of protein-mineral, protein-protein, and feedback between macromolecules and crystals [6–17]. Many studies of matrix proteins were focused on seawater mollusks, *pinctada fucata* in particular, from which a majority of proteins have been extracted and identified [15–19], while few matrix proteins from freshwater mollusk have been identified, and the mechanism associated with biomineralization remains unknown.

*Hyriopsis cumingii*, known for yielding high-quality freshwater pearls, owns a dominating position in the freshwater pearl industry. Statistics indicate that the production of freshwater pearls in China constitutes 95% of that seen throughout the world, and that *H*.*cumingii* contributes 80% of that total [20]. So far, *H*.*cumingii* matrix proteins have been primarily studied at proteomics and transcriptomics level [21–28]. Whereas, the extraction and identification of individual proteins is limited reported. A 48kDa protein was extracted from the pearl of *H*.*cumingii*, providing evidence of vaterite formation [29] and the matrix protein perlucin is reported to be involved in *H*.*cumingii* nacre formation [30]. Additionally, the *H*.*cumingii* protein silkmapin is involved in nacreous- and prismatic-layer formation [31]. Furthermore, analysis of the gene α-CA (*HcCA*) from the freshwater pearl mussel *H*.*cumingii* suggests that *HcCA* can affect shell growth [32].

In order to enhance our understanding of the molecular mechanisms underlying biomineralization, a novel gene, hic31, was extracted from *H*.*cumingii* and characterized.

## Materials and Methods

### Animals

Healthy *H*.*Cumingii*, were harvested from a mussel farm in Jinhua, Zhejiang province, China. Several glass aquariums, filled with circulating, aerated freshwater, were utilized to maintain them at 23 ±2.0°C for 1 week prior to experimentation.

### Total RNA extraction and complementary DNA (cDNA) synthesis

Various tissues (marginal mantle, velum craspedon, center mantle, gill, hepatopancres, intestine, kidney, adductor muscle, foot) were sampled and frozen immediately in liquid nitrogen. RNA from these tissues was extracted using TRIzol reagent according to manufacturer's protocol (Invitrogen, Carlsbad, CA, USA), followed by the confirmation of RNA quality (concentration, purity, and integrity) by 1.2% agarose gel electrophoresis. The first strand of cDNA was synthesized in terms of the directions of FastQuant RT Kit with gDNase (TianGen Biotech Co., LTD., Germany).

### Identification of hic31 cDNA ends and bioinformatics analysis

According to the residues of MSI 31 (“GGGGG”), a degenerate sense primer F1 (5’-GGYGGYGGYGGYGGYGGY-3’, Y = A/T/C/G) for 3’ rapid amplification of cDNA ends (RACE) was designed. Then combined with the obtained C-terminal cDNA ends from 3’ RACE, a gene-specific antisense primer R1 (5’-AGCTGGGACACAAGATGGC-3’), was synthesized for 5’-RACE. The full length of hic31 cDNA sequence was obtained by amplification performed with a SMARTER RACE cDNA Amplification kit and Advantage 2 cDNA Polymerase Mix based on the manual’s instructions (Clotech, Palo Alto, CA, USA).

Comparisons of sequence similarity were conducted using the BLAST program from GenBank (National Center for Biotechnology Information, Bethesda, MD, USA(http://www.ncbi.nlm.nih.gov/)); The hic31open reading frame and the translated amino acid sequences were predicted and acquired by ORF Finder (http://www.ncbi.nlm.nih.gov/gorf/gorf.html). The signal peptide was forecasted by SignalP 4.1 Server (http://www.cbs.dtu.dk/services/SignalP/). The physical and chemical characteristics of the predicted protein were estimated by EXPASY ProtParam (http://web.expasy.org/cgi-bin/protparam/protparam)[33]; The trans-membrane structure could be detected by TMHMM Server v.2.0 (Center for Biological Sequence Analysis, Denmark, http://www.cbs.dtu.dk/services/TMHMM/) and potential glycosylation and phosphorylation sites were analyzed using CBS prediction servers (Center for Biological Sequence Analysis, http://www.cbs.dtu.dk/). The secondary and high structure prediction was performed by accessing into Phyre^2^ (http://www.sbg.bio.ic.ac.uk/phyre/) [34]. Protein structural domains were predicted by using the Simple Modular Architecture Research Tool SMART(http://smart.embl-heidelberg.de/) [35] and PROSITE (http://prosite.expasy.org/prosite.html) [33];Through TargetP 1.1 Server (Center for Biological Sequence Analysis, Denmark, http://www.cbs.dtu.dk/services/TargetP/), the sub-cellular location of hic31 protein was estimated.

### Tissue-specific gene expression and its pattern in pearl sec during early stages of pearl formation

In order to examine the specific expression of hic31 in tissues by qRT-PCR, six individuals were sampled and cDNAs of various tissues were used as templates prepared as described at section 2.2. In addition, 45 individuals (five for each time point) were prepared for expression examination during pearl sac formation and its early development. Optimal primer pairs, which could generate single PCR product and display an amplification efficiency near the theoretical 100%, were screened out by plotting standard curves. The *EF1α* gene from *H*. *cumingii* was amplified and its expression level acted as an internal standard reference since the gene expression level was verified to be constant among all tissues [36]. qRT-PCR catalyzed by SYBR Premix ExTaq II (Tli RNaseH Plus) (Takara Bio. Inc., Japan), was then performed in triplicate for each template on the CFX96 real-time PCR Detection System (Bio-Rad, Hercules, CA, USA) in a 20μL reaction comprised of 10μL SYBR Premix Ex Taq II (Tli RNaseH Plus)(2×), 0.8μL of each primer (10μM), 1.0μL cDNA (150ng/μL), and 7.4μL RNase-free water. The program was set as follows: 95°C for 3 min, 40 cycles of 95°C for 5 s and 60°C for 30s, followed by a dissociation curve analysis of 5s per step from 65 to 95°C. The cycle threshold (Ct) values of each sample were then analyzed according to the2^−ΔΔCt^ method [37] to determine relative expression levels in different tissues against *EF1α* gene expression level in the corresponding samples.

### *In situ* hybridization of hic31 in mantle

To determine the hic31exact expression location in mantle, *in situ* hybridization was conducted. The RNA sense and antisense probes of hic31 were first synthesized by the use of T7 or SP6 RNA polymerase respectively, then a rectangular portion of fresh mantle tissue (0.8×0.5cm) was sampled and immediately fixed in 4% paraformaldehyde (freshly prepared using 0.1% DEPC water) for 6 h, followed by at least 20h incubation at 4°C in 20%-25% sucrose. Frozen sections could be prepared through the use of freezing microtome (LeicaCM 1950, Wetzlar, Germany), followed by slicing the tissue to 10μm thickness and mounting the sliced pieces on poly-lysine pretreated slides. *In situ* hybridization was carried out according to the manufacturer protocol (Enhanced Sensitive ISH Detection Kit, Boster, and Switzerland) with slight changes.

## Results

### cDNA cloning and sequence analysis

**Fig 1:**
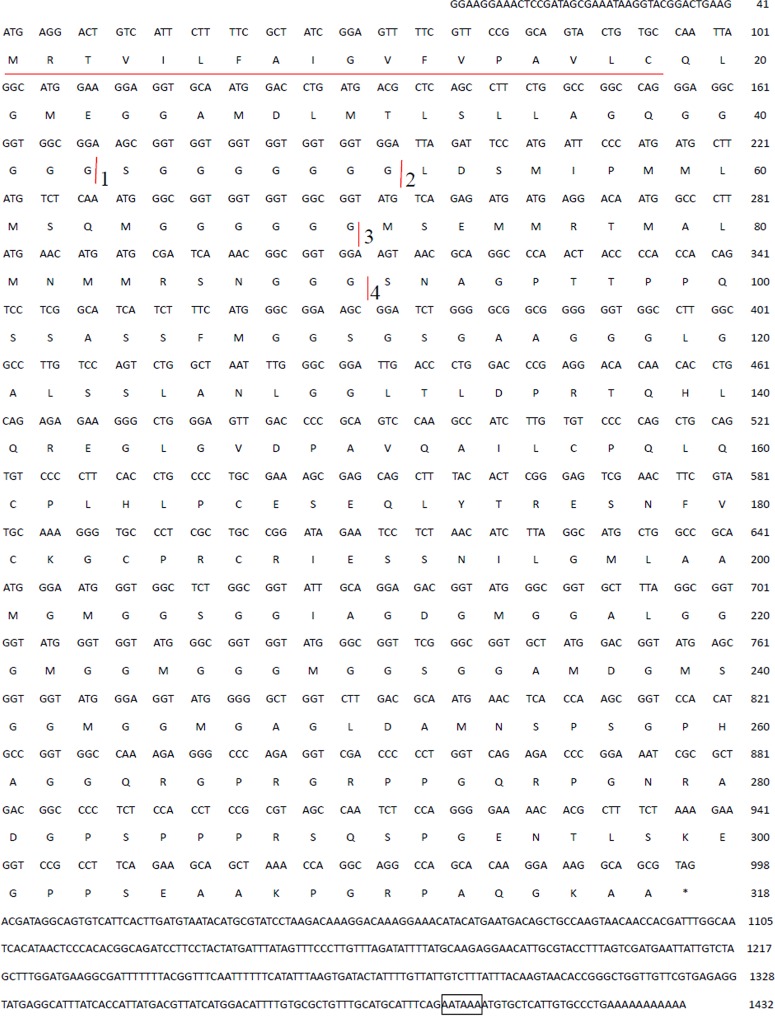
cDNA and deduced amino acid sequence of hic31. The putative signal peptide is shown underlined. The putative polyadenylation signal (AATATA) is shown underlined boxed. The cDNA sequence of hic31 has been submitted to Genebank (Accession No. KR534872).

The 3’ RACE procedure amplified 1260bp, and 5’ RACE obtained a 516bp fragment. The full 1432bp cDNA sequence (Fig 1) of hic31 was determined by combining the two fragments. Sequence analysis reveals that the open reading frame starts at ATG (position 42) and stops at TAG (position 998). The open reading frame encodes a protein of 317 amino acids, with a theoretical molecular weight of 30.7kDa. The predicted amino acids sequence contains a signal peptide from residues 1–18 (Fig 1). Without regard to the signaling peptide, the theoretical molecular weight is 28.8kDa and the isoelectric point is 7.00.

### Protein structure prediction

**Fig 2:**
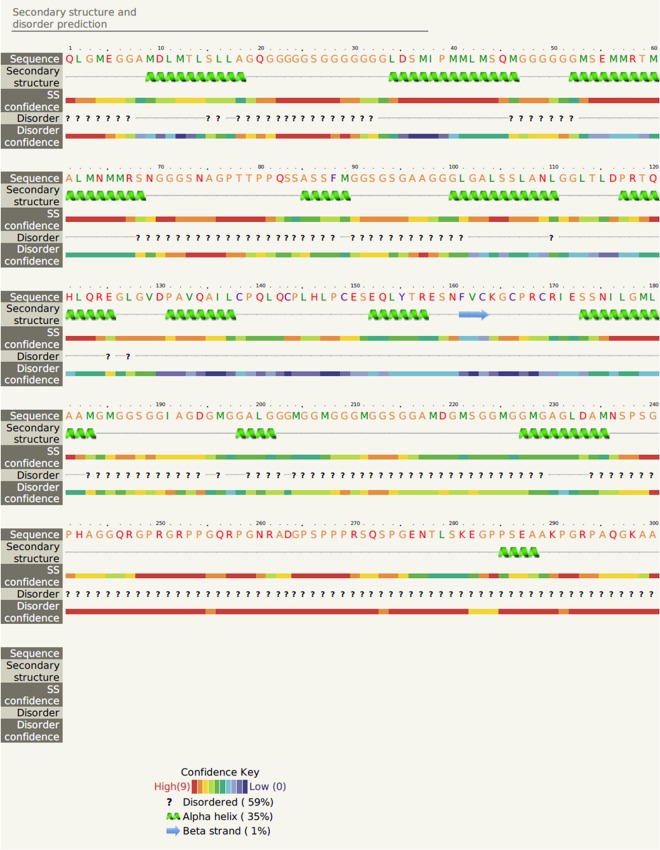
Secondary structure prediction of hic31. Based on the protein sequence of hic22, the secondary structure prediction is performed by Phyre^2^. The amino acids are colored based on the physiochemical properties of the side chains. The regions adopting putative α-helix and β-sheet conformations are represented as green spiral and blue arrow, respectively. The degrees of confidence 0.9 are also indicated by a rainbow color gradient.

**Fig 3:**
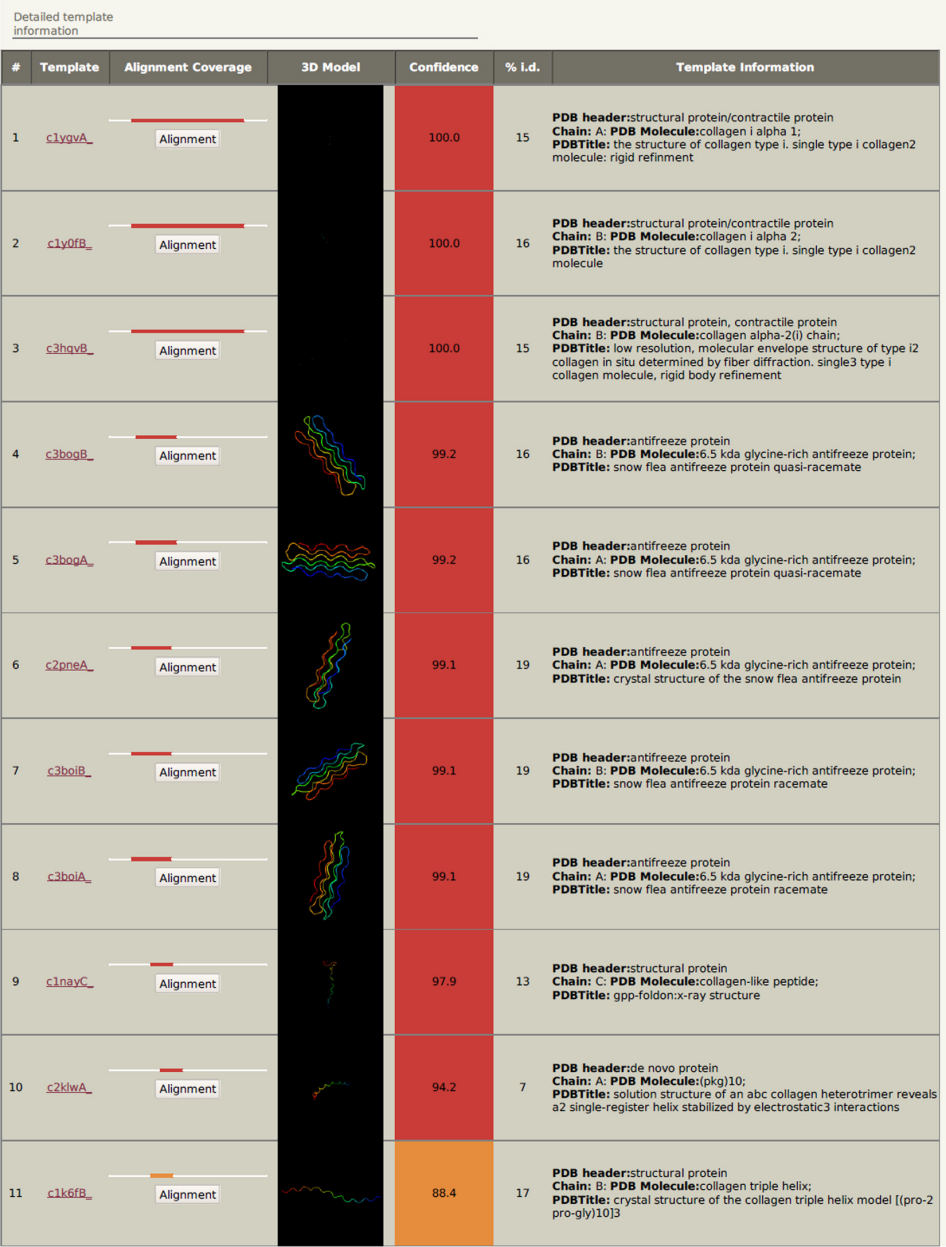
Detailed information about template in the secondary structure prediction.

**Fig 4:**
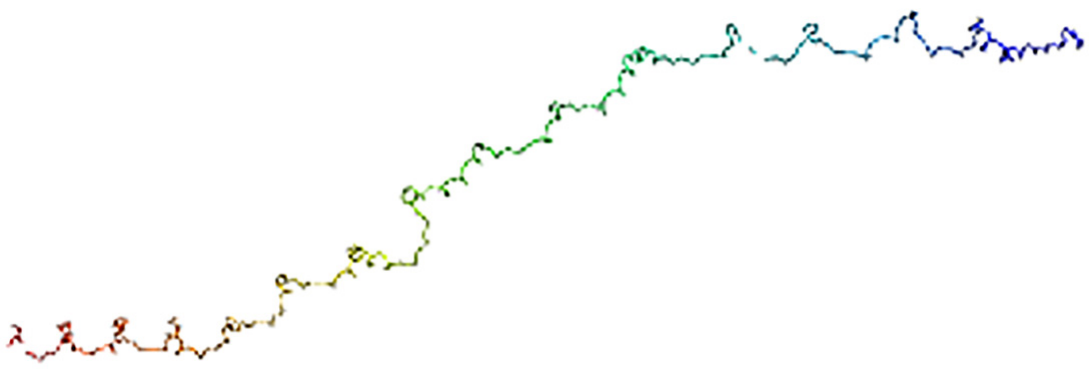
Three dimensional structure prediction of hic31. The tertiary structure prediction is performed by Phyre^2^.

Secondary structure prediction indicated that hic31 is primarily composed of α-helices (Fig 2). Although BLASTP results identified no homology with other shell matrix proteins or any other known proteins, the protein tertiary structure is similar to that of collagen, type I, alpha 1 and alpha 2 (Figs 3 and 4).

### Tissue expression and *in situ* hybridization

**Fig 5:**
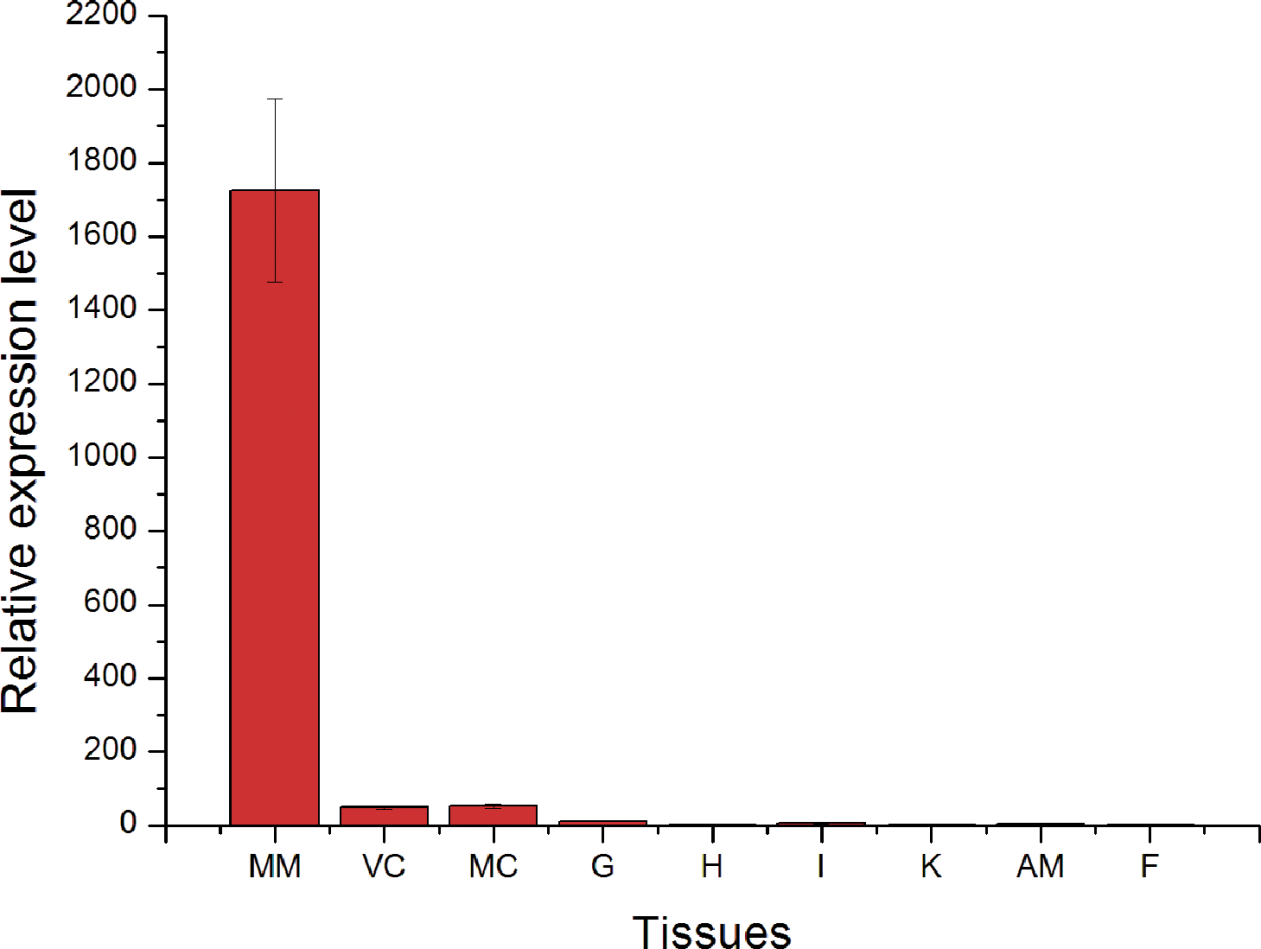
Tissue-specific expression of hic31 by qRT-PCR. MM, Marginal mantle; VC, velum craspedon; CM, Center mantle; G, gill; H, hepatopancres; I, Intestine; K, kidney; AM, adductor muscle; F, Foot.

**Fig 6:**
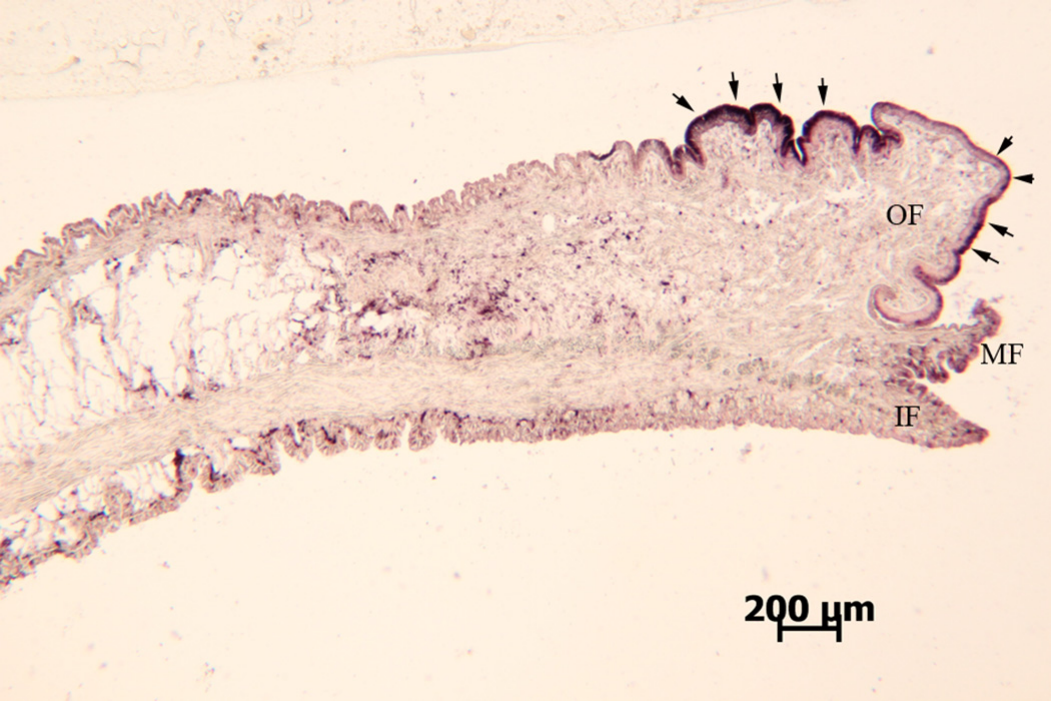
*In situ* hybridization analysis of hic31 gene expression in the mantle of *Hyriopsis cumingii*. IF, inner fold; MF, middle fold; OF, outer fold.

The hic31 expression level was detected in seven tissues (intestine, adductor muscle, foot, gill, blood, mantle edge, and pallial) by qRT-PCR. The results indicate that hic31 is specially expressed in mantle tissue, and that expression occurs primarily at the edge rather than the pallial region (Fig 5). To confirm the hic31 expression in the mantle tissue, *in situ* hybridization on frozen mantle sections using digoxigenin (DIG)-labeled hic31-specific probes were performed. The results revealed strong signals in the epithelial cells at the mantle edge (Fig 6).

### Expression pattern of hic31 during pearl sac formation and early development

The expression of hic31 in pearl sac was detected by qRT-PCR on days 3,6,9,12,19,26,33,45,and77 after insert operation of pearl tablet into *H*. *cumingii*, the time span mentioned above includes the early development of pearl sac and pearl initially biomineralization.

**Fig 7:**
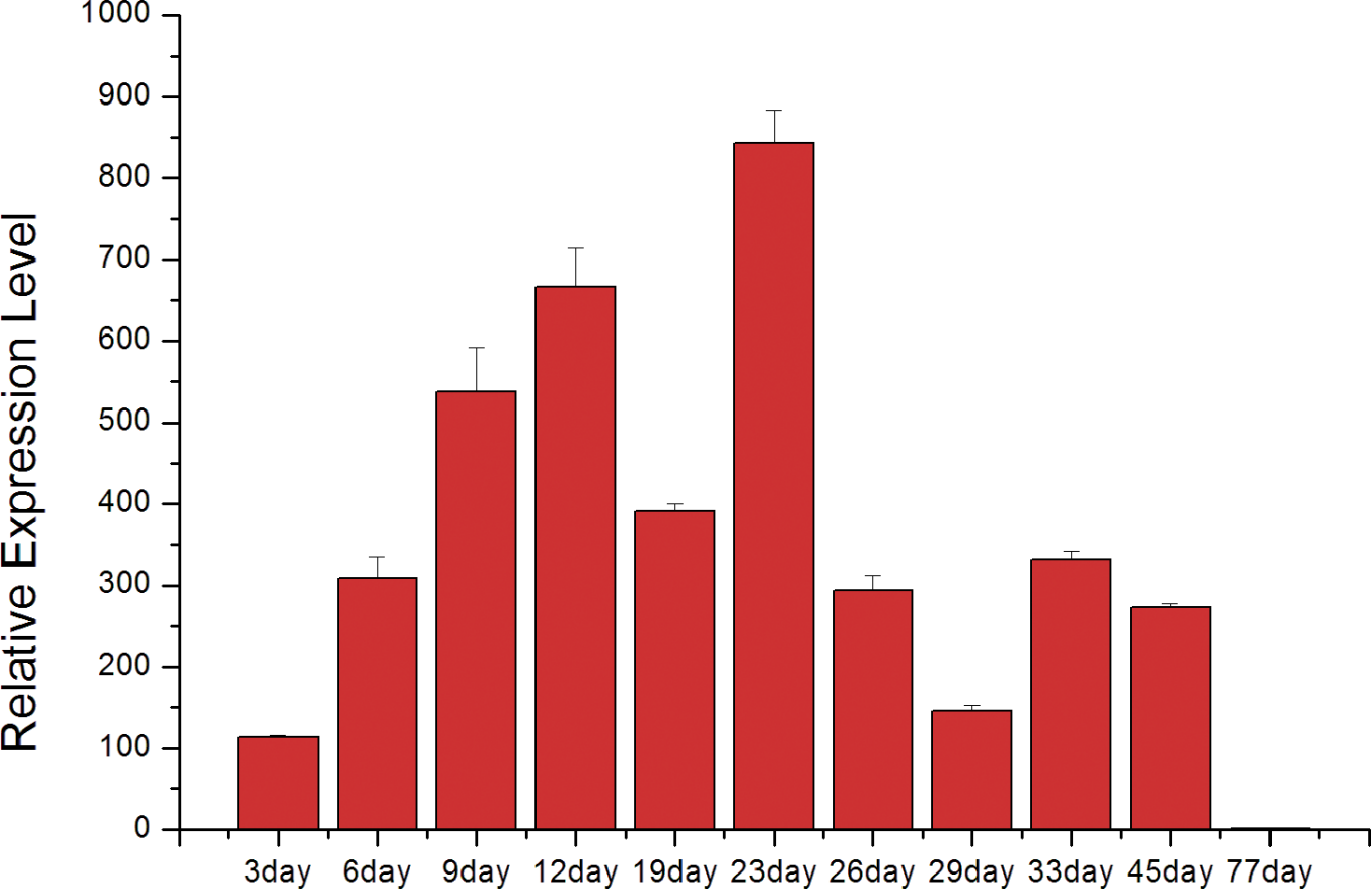
The relative expression level of hic31 in the pearl sac during the early stages of pearl formation after implantation.

Data analysis revealed that hic31 expression increased during early stages of pearl sac development between days 3–23 (Fig 7). After day 23, the expression of hic31 significantly decreased, and remained at a relatively low level until day 45. At day 77, no hic31 expression was observed.

## Discussion

A novel shell matrix protein, hic31, was identified from mantle of the freshwater mussel, *H*. *Cumingii*. Sequence composition analysis of amino acids (Table 1) revealed that it had a high proportion of glycine residues (26.67%), and glycine residues are frequently clustered as multiple polyglycine blocks ((Gly)_n_(n>2)) in the N-terminal region (residues 39–43, block 1; residues 45–51, block 2; residues 65–70, block 3; residues 88–90, block 4). Block 1 and 2 were separated by a serine (Ser).blocks 2 and 3, and blocks 3 and 4 were all subdivided by a methionine (Met)-rich region. The longer poly glycine blocks in other region (residues 201–245) are also frequently subdivided by Met or Ser. These structural characteristics result in a similar distribution pattern of Met and polyglycine blocks, leading to multiple repeats of (Gly)_m_X(Gly)_n_(m>1,n>1,where X prefers to Met or Ser). Met is hydrophobic and Ser residues have a hydroxyl group, however, there appears to be no regularity in terms of arrangement of differently-sized poly glycine blocks. In addition, the acidic amino acids, aspartic acid (Asp) and glutamic acid (Glu) always appear separately. The Asp is only surrounded by neutral amino acids while Glu is coupled with neutral or alkaline amino acids. Lysine (Lys) and proline (Pro) is primarily distributed in the C-terminal region of which Lys was presumed to initiate formation of a basic region to enhance interaction with anionic molecules during shell formation, such as CO_3_^2-^[38, 39].

**Table 1:** Amino acid composition (mole percent) of Hic31.

Amino acid	Hic31
Gly (G)	26.67%
Ser (S)	9.67%
Met (M)	9.33%
Leu (L)	8.67%
Ala (A)	8.33%
Pro (P)	8.33%
Arg (R)	4.67%
Gln (Q)	4.67%
Glu (E)	3.33%
Asn (N)	3.00%
Asp (D)	2.67%
Thr (T)	2.67%
Cys (C)	2.00%
Ile (I)	1.67%
Lys (K)	1.33%
His (H)	1.00%
Val (V)	1.00%
Phe (F)	0.67%
Tyr (Y)	0.33%

Secondary structure prediction indicates that hic31 tertiary structure is similar to that of collagen, type I, alpha 1 and alpha 2 (Fig 2), but BLASTP identified no shared homology with collagen. It may be considered that hic31 folds into a similar structure of collagen only. In vertebrates, the biomineralization of hard connective tissues, such as bone, dentin, and cementum, involves the deposition of calcium phosphate within a collagenous matrix [40, 41]. The collagen formed the basic organic frameworks (collagen fibrils) in these tissues and minerals existed both within and outside of the collagen fibrils [42, 43]. For hydroxyapatite formation, non-collagenous proteins play key roles given that collagen alone does not induce crystal formation [44–46]. This may indicate that hic31 is involved in prismatic layer biomineralization as a framework matrix protein. During the formation of prismatic layer, the organic matrix performs as an organic layer, where newly-formed crystals are embedded. Following this, the inter-prismatic organic membrane of the prismatic layer is produced by squeezing between neighboring crystals [47]. The hic31 may play key roles in this process. Secondary structure prediction also indicated structural similarities between hic31 and antifreeze protein, however, the alignment coverage between the two proteins is narrower than that observed between hic31 and collagen (Fig 3).

Quantitative analysis of *H*. *cumingii* hic31 expression performed on tissues by qRT-PCR indicated that hic31 is specially expressed in marginal mantle. To determine a more precise expression site of hic31 in the mantle edge, *in situ* hybridization signals were detected on frozen mantle sections. Strong signals were detected in the dorsal epithelial cells of the outer fold at the mantle edge, and weak signals were detected in inner epithelial cells of the outer fold. These results indicate that hic31 is a prismatic layer matrix protein.

The expression of hic31 during early pearl sac development increased significantly during early stages, and decreased obviously following day 23 until no expression was detected on day 77. From previous studies [30, 31, 48], the first nacreous layer has been formed on day 23. The CaCO_3_, first deposited at the nucleus of calcitic prismatic layer found in the pearl cross-section, followed by nacreous layer formation on the prismatic layer [49, 50]. Therefore, the increased hic31expression from day 3 through day 19 may be responsible for prismatic layer biomineralization, and the period from day 19 to day 23 is a transition time from prismatic layer to nacreous layer biomineralization. Besides, the expression of hic31 decreased significantly after day 23, and there was no measureable expression observed when the manner of the nacreous layer biomineralization remains mature and steady. These data suggest that hic31 may play important roles in pearl prismatic layer formation.
